# 5-HT_2A_ Receptor Binding in the Frontal Cortex of Parkinson's Disease Patients and Alpha-Synuclein Overexpressing Mice: A Postmortem Study

**DOI:** 10.1155/2016/3682936

**Published:** 2016-08-04

**Authors:** Nadja Bredo Rasmussen, Mikkel Vestergaard Olesen, Tomasz Brudek, Per Plenge, Anders Bue Klein, Jenny E. Westin, Karina Fog, Gitta Wörtwein, Susana Aznar

**Affiliations:** ^1^Research Laboratory for Stereology and Neuroscience, Bispebjerg and Frederiksberg Hospitals, Copenhagen University Hospital, 2300 Copenhagen, Denmark; ^2^Department of Neuroscience and Pharmacology, University of Copenhagen, 2200 Copenhagen, Denmark; ^3^Department of Drug Design and Pharmacology, University of Copenhagen, 2200 Copenhagen, Denmark; ^4^Department of Neurodegeneration, Lundbeck A/S, Ottiliavej 9, 2500 Valby, Denmark; ^5^Laboratory of Neuropsychiatry, Department of Neuroscience and Pharmacology, University of Copenhagen and Mental Health Center Copenhagen, 2200 Copenhagen, Denmark

## Abstract

The 5-HT_2A_ receptor is highly involved in aspects of cognition and executive function and seen to be affected in neurodegenerative diseases like Alzheimer's disease and related to the disease pathology. Even though Parkinson's disease (PD) is primarily a motor disorder, reports of impaired executive function are also steadily being associated with this disease. Not much is known about the pathophysiology behind this. The aim of this study was thereby twofold: (1) to investigate 5-HT_2A_ receptor binding levels in Parkinson's brains and (2) to investigate whether PD associated pathology, alpha-synuclein (AS) overexpression, could be associated with 5-HT_2A_ alterations. Binding density for the 5-HT_2A_-specific radioligand [^3^H]-MDL 100.907 was measured in membrane suspensions of frontal cortex tissue from PD patients. Protein levels of AS were further measured using western blotting. Results showed higher AS levels accompanied by increased 5-HT_2A_ receptor binding in PD brains. In a separate study, we looked for changes in 5-HT_2A_ receptors in the prefrontal cortex in 52-week-old transgenic mice overexpressing human AS. We performed region-specific 5-HT_2A_ receptor binding measurements followed by gene expression analysis. The transgenic mice showed lower 5-HT_2A_ binding in the frontal association cortex that was not accompanied by changes in gene expression levels. This study is one of the first to look at differences in serotonin receptor levels in PD and in relation to AS overexpression.

## 1. Introduction

Parkinson's disease (PD) is clinically characterized by motor symptoms consisting of bradykinesia, resting tremor, rigidity, and postural instability. One of the leading hypotheses for PD pathogenesis focuses on alterations in alpha-synuclein (AS) expression, neuronal accumulation, and aggregation of AS—including formation of Lewy bodies—as a main causative factor in the pathological cascade [1]. Though PD principally is classified as a movement disorder, it has now become recognised that PD features a complex burden of different motor and nonmotor symptoms (NMS) [1, 2]. NMS covers a range of symptoms including hyposmia, visual hallucinations, sleep disturbances, a variety of dysautonomic symptoms, depression and other mood disorders, and impairment of cognition and consequently affected executive function [3].

The key brain area involved in cognition and executive function is the prefrontal cortex (PFC). The serotonin 5-HT_2A_ receptor is highly expressed in PFC areas, playing an important role in executive function [4] and in modulating the cognitive control of our emotional responses during decision-making [5], making them essential for inhibitory response control, which recently has been shown to be impaired in PD patients [6]. Alterations in cortical 5-HT_2A_ receptor levels have been reported in Alzheimer's disease patients [7]. We have shown how this is associated with beta-amyloid accumulation in transgenic mice models of beta-amyloid overexpression [8]. Here we wanted to investigate whether this could also be the case for PD.

The aim of this study was, on one side, to look for 5-HT_2A_ receptor alterations in the frontal cortex of postmortem brain tissue from PD patients and, secondly, to investigate by use of transgenic mice whether overexpression of AS would lead to alterations in frontal cortical 5-HT_2A_ receptors. Alterations in serotonergic innervation have previously been described in relation to PD [9], but information is lacking about the extent to which serotonin receptors, and more specifically 5-HT_2A_ receptors in PFC, are affected in this disease.

For the first approach, we used membrane suspensions of human frontal cortex tissue of PD and control brains in order to perform receptor binding studies with the 5-HT_2A_-specific radioligand, [^3^H]-MDL 100907, and furthermore measured AS protein levels by western blotting. Second, we used a transgenic mouse model overexpressing human AS to examine region-specific 5-HT_2A_ receptor changes by autoradiography analysis of [^3^H]-MDL 100.907 binding, followed by gene expression analysis.

## 2. Materials and Methods

### 2.1. 5-HT_2A_ Receptor Binding Assay in Human Frontal Cortex Tissue

Postmortem frontal cortex brain tissue (BA9 region) from PD patients and controls, deceased from nonneurological causes, was used for the binding assay. All brain samples were fresh-frozen, stored at minus 80°C until further use. Brains were acquired from the Harvard Brain Tissue Resource Center, USA, and the Brain Bank at Bispebjerg University Hospital (Copenhagen, Denmark) (BBH-2010-06, I-suite number 00971). The initial total number of samples was *n* = 8 for PD and *n* = 7 for controls, but one sample from each group was excluded from further analysis as they showed significance in a Grubbs outlier test. The PD and control samples included in the statistical analysis are listed in Table 1.

Brain tissue samples from PD patients were provided by the Harvard Brain Tissue Resource Center, USA. The mean age at death for the patients was 75.4 years and mean postmortem index (PMI)—time from death to autopsy—was 20.2 hours. Brain tissue samples for the controls were provided by the Brain Bank of the Research Laboratory for Stereology and Neuroscience, Bispebjerg University Hospital, Denmark. The causes of death in the control group were gastrointestinal (GI), cardiopulmonary (CP), and/or cancer related, and mean age at death was 68.5 years and mean PMI was 43.0 hours. There was no significant difference between the PD and control group in terms of age of death (*Student's t*-*test*, *p* > 0.05) and gender distribution (chi-squared test, *p* > 0.05), while there was a significant difference in PMI (*Student's t*-*test*, *p* < 0.05).

For the brain membrane preparation, 450 mg frontal cortex tissue of each sample was homogenized in HEPES-buffer (25 mM HEPES, 120 mM NaCl, 5 mM KCl, 1.2 mM CaCl_2_, and 1.2 mM MgSO_4_, pH = 7.5). The homogenate was spun down for 5 minutes, resuspended, and spun down again for 10 minutes at 4700 rpm and again resuspended in buffer in order to obtain a 5% membrane suspension (MS). Three aliquots of the MS were stored at minus 20°C until further use. Three independent but identical saturation binding assays were performed on three different days for each sample. Brain samples were blinded. For each assay, 100 *μ*L of the MS was added to each well in a 24-well plate together with 100 *μ*L [^3^H]-MDL 100.907 (at six different concentrations: 0.14; 0.28; 0.53; 1.07; 2.12; 4.63 nM) and HEPES-buffer until reaching a total volume of 300 *μ*L. For the nonspecific binding (NSB) 10 *μ*M unlabelled MDL 100.907 was added to each of the concentrations. Three total binding (TB) and NSB measurements were obtained for each concentration of [^3^H]-MDL 100.907. Incubation time was 1 hour at room temperature.

Before filtration 1 mL ice-cold HEPES-buffer was added to each well. Membranes were filtered on printed Filtermat B90–120 mm from Wallac covered with polyethyleneimine (PEI) 0.05% using a Connectorate filtration machine fitted with a 24-pin head (*Connectorate AG*,* Dietikon, Switzerland*). The filters were washed in ice-cold HEPES-buffer for 30 seconds and left to dry on a heating plate at 98°C. Once dried, filters were covered with MeltiLex scintillation gel and counted on the scintillation counter (6-channel micro beta counter, Perkin Elmer). The three measurements for each concentration point were averaged into one measurement and specific binding (SB) was determined as the difference between TB and NSB and plotted against ligand concentration in GraphPad. *K*_*d*_ and *B*_max_ values for each sample were calculated according to a nonfit linear analysis curve. Protein concentration was determined from wet weight.

### 2.2. AS Protein Levels in Human Frontal Cortex Tissue Measured by Western Blotting

For the tissue lysate preparation, 50 mg of tissue was dissected out from the 7 PD and 6 normal controls brains from equivalent area as above and placed immediately in ice-cold Tissue Extraction Reagent II (Invitrogen, Thermo Fisher Scientific, Waltham, MA, USA) containing protease inhibitor cocktail (Sigma-Aldrich, St. Louis, MO, USA). The tissue samples were homogenized using the MagNA Lyser Instrument (2x 6000 rpm, 25 s) and related MagNA Lyser Green Beads (Roche), in an appropriate volume of tissue extraction reagent (10 mL/g). The homogenates were incubated on ice for 10 minutes and centrifuged at 16 000 ×g for 20 minutes at 4°C. The supernatants were collected, separated into aliquots, and stored at minus 80°C until use. A portion of the supernatant was reserved for protein determination using the Bradford reagent (Sigma-Aldrich) and subsequent measurement of absorbance was done at 595 nm using bovine serum albumin as standard.

Next, samples were prepared for electrophoresis by diluting each 20 *μ*g of protein lysate with a 4x NuPAGE® sample buffer containing 2 mM of cross-linker dithiobis[succinimidylpropionate] (DSP) (Pierce, Thermo Fisher Scientific, Waltham, MA, USA). DSP was dissolved in dimethyl sulfoxide to a 50x stock concentration prior to addition to the protein samples. Samples were incubated with cross-linker for 30 minutes at 37°C, followed by addition of 5%  *β*-mercaptoethanol (*β*ME) and incubation at 70°C for 10 minutes. The application of reducible amine-reactive cross-linker DSP followed by reductive cleavage (5%  *β*ME) prior to sodium dodecyl sulfate polyacrylamide gel electrophoresis and electroblotting improves significantly immunodetection of alpha-synuclein monomers [10].

The samples were electrophoresed on NuPAGE 4–12% Bis-Tris Gels with NuPAGE 2-(N-morpholino)ethanesulfonic acid-sodium dodecyl sulfate (Life Technologies) running buffer and Novex® Sharp Pre-Stained Protein Standard (Life Technologies). As standards, we used 10 *μ*g of human recombinant full-length alpha-synuclein (rPeptide) treated and loaded on gels simultaneously with the tissue lysates. After electrophoresis, gels were blotted onto Odyssey® nitrocellulose membranes 0.22 *μ*m (LI-COR Biosciences, Cambridge, UK) using the semidry Bio-Rad apparatus (Bio-Rad Laboratories, Hercules, CA, USA) for 60 minutes, using a 200 mA/membrane constant current in NuPAGE transfer buffer (Life Technologies) containing 20% methanol. Posttransfer membranes were treated with 0.4% paraformaldehyde in phosphate-buffered saline (PBS) for 30 minutes at 21°C, rinsed with Milli-Q water, and then blocked in Odyssey blocking buffer (PBS) (LI-COR) for 1 hour at 21°C. Treatment with low concentrations of paraformaldehyde prevents washing off alpha-synuclein from nitrocellulose membranes [11].

Blots were then incubated overnight at 4°C with rabbit monoclonal anti-alpha-synuclein antibody MJFR1 (Abcam # ab138501, 1 : 5000) in Odyssey blocking buffer with 0.1% Tween-20. All membranes were at the same time also incubated with an anti-GAPDH antibody (clone FL-335) (# SC-25778, 1 : 1000; Santa Cruz Biotechnology, Santa Cruz, CA, USA) as a loading control. Membranes were then washed 3x 15 minutes in PBS with 0.1% Tween-20 and incubated in secondary antibody IRDye® 800CW Goat anti-rabbit IgG (# 926-32211; LI-COR Biosciences) 1 : 15 000 and IRDye 680LT Goat anti-mouse IgG_1_-specific (# 926-68050; LI-COR Biosciences) 1 : 20 000 in PBS + 0.1% Tween + 0.01% sodium dodecyl sulfate at 21°C in the dark. Subsequently, the membranes were washed 3x 15 minutes in PBS, rinsed in Milli-Q water, air dried, and developed on LI-COR Bioscience Odyssey 9120 Infrared Imaging System.

Scanned western blots were analyzed with Image Studio Lite software v.5.2 (LI-COR Biosciences). The infrared signals after background subtraction were normalized to the loading control signal.

### 2.3. Transgenic Animals, h-SNCA Mice, Overexpressing Human Alpha-Synuclein

Mice used in the study were male, 52-week-old, transgenic (tg) F28SNCA mice on a C57BL/6 background (*n* = 10) overexpressing wild-type human SNCA (an AS coding gene) under the control of mouse AS promoter [12, 13] and C57BL/6 wild-type (wt) mice (*n* = 10) (Taconic, H. Lundbeck A/S, Denmark). All animal experiments were approved by the Danish Veterinary and Food Administration (DVFA) and were conducted in accordance with European standards on animal welfare.

### 2.4. Ligands

The 5-HT_2A_ receptor antagonist, [^3^H]-MDL 100.907, [R-(+)-*α*-(2,3-dimethoxyphenyl)-1-[2-(4-fluorophenyl)-ethyl]-4-piperidine-methanol], was synthesized and radiolabelled by NOVANDI Chemistry AB, Sweden. The specific activity of [^3^H]-MDL 100.907 was determined to be 75.676 Ci/mmol (2.8 Tbq/mmol). Radiochemical purity was greater than 97%.

### 2.5. 5-HT_2A_ Receptor Autoradiography

Mice were decapitated and brains rapidly removed and frozen on powdered dry ice and kept at minus 80°C until sectioning. Prefrontal cortex regions were cut into 15 *μ*m coronal sections on a cryostat, thaw-mounted onto Superfrost Plus slides, and stored at minus 20°C until further processing.

Autoradiography was performed using 1 nM [^3^H]-MDL 100.907 for 5-HT_2A_ receptor TB, with addition of 10 *μ*M ketanserin to determine NSB. Briefly, sections were thawed at room temperature for a minimum of 30 minutes prior to the experiment. For preincubation, sections were submerged in 5°C tris-buffer (50 mM tris-HCl buffer, pH = 7.4, with 0.01% ascorbic acid) for 15 minutes—with or without ketanserin. Sections were incubated for 120 minutes at 5°C in tris-buffer with the ligand, with or without ketanserin. Concentrations of radioligand were determined using a scintillation counter. After incubation, slides were dipped in ice-cold tris-buffer for 5 seconds. Slices were then washed again 2x 15 minutes in ice-cold tris-buffer and dipped in ice-cold dH_2_O for 20 seconds. Sections were left to dry under a fume hood overnight. After drying, sections were exposed for 5 weeks to a BAS IP-TR2040 Fuji Imaging Plate (Science Imaging, Sweden) together with [^3^H] specific microscales at 4°C. Imaging plates were scanned on the BAS-2500 Phosphor Image Scanner: FLA-9000, Starion. ImageJ was used for densitometric analysis of signal intensity. Concentrations were expressed as nCi/mg. Quantification was performed blindly in separate regions of interest (ROIs) according to Paxinos and Franklin's atlas of the mouse brain [14].

### 2.6. RT-qPCR Quantification of 5-HT_2A_-R Expression

RNA was extracted from fresh frontal cortex sections that were scraped off from parallel slides to the ones allocated for the receptor autoradiography analysis. For each animal, a total of 12 coronal frontal sections of 15 *μ*m thickness were pooled together. Total RNA was purified using the PureLink RNA Mini Kit (Ambion) following the manufacturer's instructions. All RNA samples were treated once with DNase using TURBO DNA-free Kit (Ambion) according to the manufacturer's instructions. RNA samples were suspended in RNase-free water and were quantified using Agilent 2100 Bioanalyzer (Agilent Technologies). Only samples with RIN ≥ 6 were included in the analysis. RNA samples were stored at minus 80°C until further use.

A two-step real-time PCR was subsequently performed: RNA samples matched on concentration were reverse transcribed into cDNA with qScript cDNA SuperMix Kit (Quanta BioSciences) according to manufacturer's instructions. SuperMix reaction mixture consisted of 5x reaction buffer containing optimized concentrations of MgCl_2_, dNTPs (dATP, dCTP, dGTP, and dTTP), recombinant RNase inhibitor protein, qScript reverse transcriptase, random primers, oligo(dT) primers, and stabilizers. cDNA synthesis step was performed following incubation times of 5 minutes at 25°C, 30 minutes at 42°C, 5 minutes at 85°C, and 5 minutes at 25°C. Thereafter cDNA product was diluted 5-fold in nuclease-free water and kept at minus 20°C until further use.

To check RNA samples for contamination with nuclear DNA a negative control PCR was done on all samples prior to cDNA synthesis omitting the reverse transcription step.

Real-time PCR reactions were performed using PerfeCTa SYBR Green FastMix (2x) Kit (Quanta BioSciences) with forward and reverse primers specific for the 5-HT_2A_ receptor (mouse), Rpl13a (mouse reference gene), and GAPDH (mouse reference gene) (TAG Copenhagen). Primer sequences were as follows: 5-HT_2A_: F = 5′-GCA GTC CAT CAG CAA TGA GC-3′ and R = 5′-GCA GTG GCT TTC TGT TCT CC-3′; Rpl13a: F = 5′-GGA GGG GCA GGT TCT GGT AT-3′ and R = 5′-TGT TGA TGC CTT CAC AGC GT-3′; GAPDH: F = 5′-CAT CAA GAA GGT GGT GAA GCA-3′ and R = 5′-CTG TTG AAG TCA CAG GAG ACA-3′. qPCR was performed with 30 seconds for initial activation at 95°C, then 40 cycles of 5 seconds at 95°C for denaturation, 15 seconds at 60°C for annealing (END read), and 10 seconds at 72°C for elongation. To verify product a melting curve analysis between 55 and 95°C was performed after each run. Relative quantification (target/reference) was made using the ΔCt method [15].

### 2.7. Statistical Analyses

All statistical analyses were performed in GraphPad Prism 6. Significance level was set at a *p* value ≤ 0.05. All values are presented as mean ± standard error of the mean (SEM).* Student's t*-*test* was used for comparing mRNA levels for the receptors analyzed, AS protein levels, and *K*_*d*_ and *B*_max_ values between the PD and control brain samples. Multiple *t*-tests were used for comparing receptor binding levels in h-SNCA tg and wt mice for the different ROIs.

## 3. Results

### 3.1. Higher 5-HT_2A_ Receptor Binding in Postmortem Prefrontal Cortex of PD Patients

**Figure 1:**
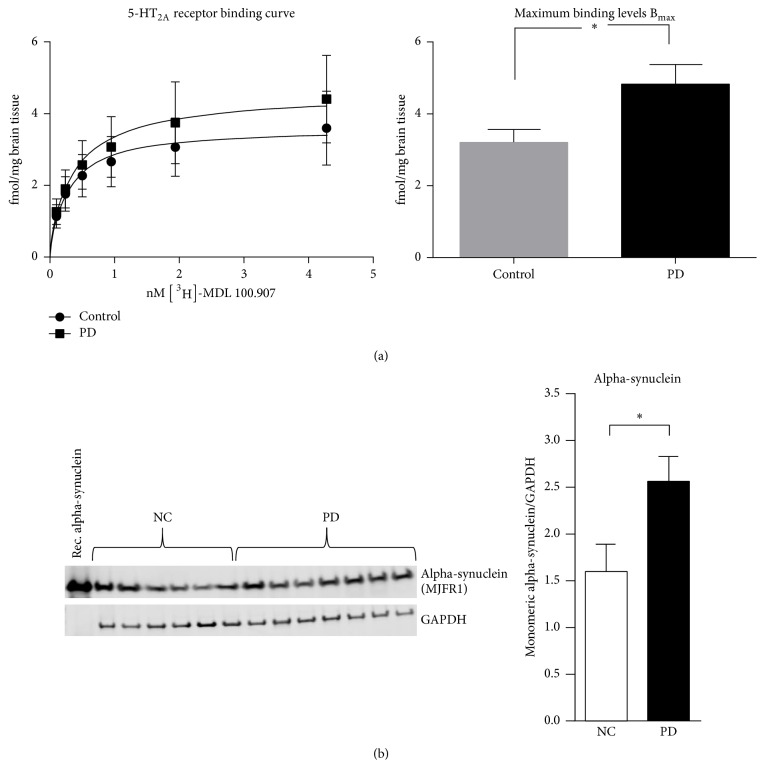
Receptor binding analysis and alpha-synuclein levels in postmortem prefrontal cortex of Parkinson's disease (PD) patients. (a) Binding assays with [^3^H] MDL100.907 comparing membrane suspensions from postmortem prefrontal cortex of Parkinson's disease (PD) patients and controls show higher maximum binding levels (*B*_max_) of the 5-HT_2A_ receptor in the PD group. (b) Western blot showing higher levels of alpha-synuclein proteins in the PD brains included in the binding assay. Control = normal controls; PD = Parkinson's disease patients. Significant ^*∗*^ *p* < 0.05.* Student's t*-*test* with mean ± SEM.

The binding assay, performed on human brain tissue from the frontal cortex, showed a significant difference in *B*_max_ of the 5-HT_2A_ receptor between PD (*n* = 7) and control (*n* = 6) brain samples, with higher maximum binding level in the PD group (PD: 5.7 ± 1.0; controls: 3.2 ± 0.35; *Student's test* *p* < 0.05) (Figure 1(a)). There was no significant difference between the PD and control group when comparing *K*_*d*_ (affinity) (PD: 0.5 ± 0.07; controls 0.3 ± 0.05; *p* > 0.05). Mean PMI (postmortem interval) was different between the two groups (PD and controls). We controlled for a potential effect of the PMI on binding results, and no correlation between *B*_max_ and PMI (*r* = −0.52, *p* > 0.05) or between *K*_*d*_ and PMI (*r* = −0.32, *p* > 0.05) was found.

### 3.2. Increased AS Protein Levels in the Prefrontal Cortex Region of PD Brains

Detergent soluble monomeric AS protein levels, as measured by electrophoresis and immunoblotting, were increased in the PD brains compared to controls (PD: 2.56 ± 0.27; controls: 1.56 ± 0.3;* Student's test p* < 0.05) (Figure 1(b)). No correlation was observed between AS protein and 5-HT_2A_ receptor binding levels in the PD brains (Spearman *r* = −0.48; *p* > 0.05).

### 3.3. Region-Specific Differences in 5-HT_2A_ and 5-HT_1A_ Receptor Binding in AS Overexpressing Mice

**Figure 2:**
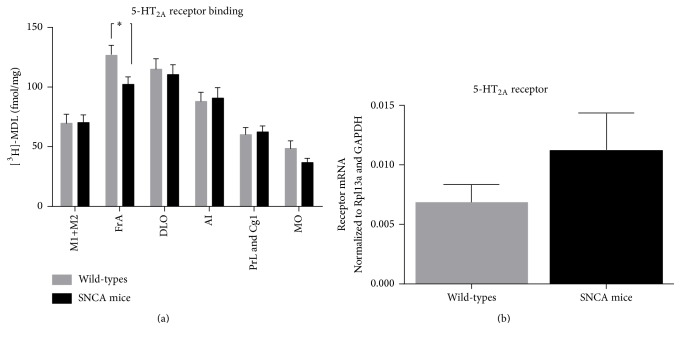
5-HT_2A_ receptor mouse prefrontal cortex autoradiography binding analysis and gene expression levels. (a) Results from [^3^H] MDL 100.907 (5-HT_2A_) binding. FrA [^3^H] MDL 100.907 binding is significantly lower in alpha-synuclein overexpressing (SNCA) mice compared to wt mice. Bars: grey: wild-type mice (*n* = 9); black: SNCA mice (*n* = 10). ^*∗*^ *p* < 0.05. Multiple *t*-test. Data illustrated with mean ± SEM. (b) Results from RT-qPCR show CT ratios (calculated using CT-ratio (receptor/reference) = 2^(CT  (reference)  −  CT  (receptor))^) comparing alpha-synuclein overexpressing (SNCA) and wt mice. Reference genes are Rpl13a and GAPDH. No difference in receptor gene expression was found. Bars show mean ± SEM.

The regions of interest (ROIs) included FrA (frontal association cortex), PrL + Cg1/2 (prelimbic cortex and cingulate cortex), MO (medial orbital cortex), DLO (dorsolateral orbital cortex), M1 and M2 (primary and secondary motor cortex), and AI (agranular insular cortex). Results from the [^3^H]-MDL 100.907 autoradiography binding analysis showed significantly lower 5-HT_2A_ receptor binding in FrA of h-SNCA mice (h-SNCA: 102.2 ± 6.5 fmol/mg; wt 126.7 ± 8.0 fmol/mg; *p* < 0.05). There were no significant differences in 5-HT_2A_ receptor binding in the other ROI measured (Figure 2(a)).

### 3.4. No Difference in 5-HT_2A_ Receptor Gene Expression Levels

Results from RT-qPCR show no difference in 5-HT_2A_ (h-SNCA: 0.01 ± 0.003; wt 0.007 ± 0.001; *p* > 0.05) receptor gene expression in the frontal cortex of h-SNCA mice when compared to wt mice (Figure 2(b)).

## 4. Discussion

In this study we looked at alterations in 5-HT_2A_ receptor binding levels in the frontal cortex from PD patients, and using a tg h-SNCA mouse model, we furthermore looked for whether AS overexpression could be associated with changes in 5-HT_2A_ receptor binding and gene expression.* In vitro* receptor binding studies on postmortem brain tissue from PD patients with increased AS levels indeed revealed significantly higher 5-HT_2A_ receptor binding levels, as indicated by higher maximum binding (*B*_max_) in the PD brains. Results obtained using the well validated human AS overexpressing mouse model [12, 13] showed lower 5-HT_2A_ receptor binding levels in FrA cortex in the h-SNCA transgenic mice. Differences in 5-HT_2A_ receptor binding levels were not accompanied by decreased gene expression.

To our knowledge this is one of the first studies directly looking at 5-HT_2A_ receptor binding levels in postmortem tissue samples from the frontal cortex of PD brains in relation to Parkinson's disease associated pathology of AS overexpression. Previously, at least two studies had addressed the question of 5-HT_2A_ receptor alterations in the frontal cortex of PD brains. A study from 1998 examined postsynaptic 5-HT_2A_ receptors in the neocortex of eight PD patients [16]. It too found an increase in 5-HT_2A_ receptor binding in PFC of postmortem PD tissue, using ligands other than those in this study (8-OH-DPAT and ketanserin). More recently, a Single-Positron-Emission-Tomography (SPECT) study, using the [^123^I]-5-I-R91150 ligand, reported lower 5-HT_2A_ receptor binding in the anterior striatum and premotor cortex and increased 5-HT_2A_ receptor binding in the occipital cortex of untreated PD patients [17]. However, no significant differences in 5-HT_2A_ receptor binding were detected in the prefrontal and parietal cortices. This discrepancy with our findings could be explained by the fact that patients included in the latter study were in early stages of the disease and therefore not directly comparable to our patient group consisting of end stage PD patients. According to Braak staging of PD, the raphe nuclei, the origin of the serotonergic projection to the substantia nigra, striatum, globus pallidus, subthalamic nucleus, thalamus, and neocortex with connections to cortical regions including frontal cortex and PFC areas—core structures of the cortico-basal ganglia-thalamo-cortical loop compromised in PD [9]—become increasingly affected in stage 2 and are completely affected in stage 3 [18, 19]—hence, the serotonergic system is mostly affected at later stages of the disease. Another explanation could be that, contrary to the study by Melse et al. [17], which included unmedicated patients, we cannot exclude an effect of the medical treatment on the receptor upregulation observed in our PD patients.

In addition to higher binding levels of 5-HT_2A_ receptor in the frontal cortex of PD brains, our study found higher concentration of AS in PD compared to controls, which corresponds well with the view that accumulation of AS plays a significant role in PD pathology. Overall, it is important to take into consideration that both in the study by Melse et al. [17] and Chen et al. [16] and in our study relatively small sample sizes were used with risk of inconclusive results. With that in mind, we can nevertheless agree that the joint results point towards disease related changes in 5-HT_2A_ receptor levels that could be associated with some of the cognitive and executive dysfunction seen in PD. More studies are needed to pursue this idea further.

In the second part of our study, our aim was to investigate whether alterations in 5-HT_2A_ receptor binding and expression could be associated with AS overexpression. Here decreased binding levels of 5-HT_2A_ receptor were found in FrA in AS overexpressing mice. This region is part of the PFC area, and even though its exact function is still unknown, FrA is most likely involved in some of the cognitive processes related to executive function. The FrA has been proposed in a human study to be important for go/no-go task performance [20]. The go/no-go task measures attention and response inhibition control [21], and interestingly these are functions impaired in newly diagnosed drug naïve PD patients [6]. The differences in receptor binding levels observed in the AS overexpressing mice were not accompanied by differences in gene expression. The lower receptor binding in the h-SNCA mice could therefore be due to regulatory effects at the posttranslational level.

Why the changes in 5-HT_2A_ receptor binding levels display opposite directions in the PD brains and in the transgenic animal model of AS overexpression is puzzling. In the mouse model we isolate a specific segment of the underlying pathology behind PD—that is AS overexpression. In the human tissue all aspects of PD pathology along with AS overexpression, that is, dopaminergic degeneration, play in, thus adding to the complexity of mechanisms that may influence the 5-HT_2A_ receptor. Striatal degeneration also results in decreased 5-HT_2A_ binding in PFC, as seen in a 6-hydroxydopamine-induced parkinsonian rat model [22]. Changes in 5-HT_2A_ receptor levels are probably the result of a more multifaceted pathology behind PD, not directly related to AS overexpression. We cannot exclude that the receptor upregulation observed in the PD brains can also be due to compensatory drug effects administered to this group of patients, that is, antidepressants or antipsychotics; as we do not have access to this information from the patient material used for this study we cannot determine this.

In summary, in PD brains we find higher levels of 5-HT_2A_ receptor binding together with higher levels of AS. An association is not supported by transgenic mouse model results, thus pointing towards a more complex and multidimensional explanation for 5-HT_2A_ changes found in PD. Nevertheless, the most important finding from the present study is that the 5-HT_2A_ receptor seems to be dysregulated in PD. Progress in the area of 5-HT_2A_ targeting treatment is already being manifested in management of PD psychosis for example [23]. Addressing this receptor in future studies is relevant for understanding and treating some of the cognitive and executive dysfunction seen in PD.

## Figures and Tables

**Table 1:** Overview of postmortem brain tissue samples used in binding assay analysis showing sample number, diagnosis/cause of death (COD) (PD patients or controls dead of other nonneurological causes), age at time of death (age (death)), gender, and PMI (postmortem interval). CP = cardiopulmonary and GI = gastrointestinal.

Parkinson's disease (PD) frontal cortex samples				Controls frontal cortex samples			
Diagnosis/COD	Age of death	Gender	PMI^*∗*^	Diagnosis/COD	Age of death	Gender	PMI^*∗*^
PD	71	M	23,42	CP	59	M	61
PD	75	M	26,06	GI	81	F	35
PD	84	F	18,92	CP	58	M	43
PD	77	M	11,23	GI	74	F	24
PD	70	M	24,12	GI	70	F	72
PD	66	M	11,21	GI/cancer	69	M	23
PD	85	F	26,25				

Mean	75,4	5 M/2 F	20,2	Mean	68,5	3 M/3 F	43

^*∗*^PMI = postmortem interval: time from death to tissue sample collection.
